# Supplementary material for: Contemporary Status of Acute Myocardial Infarction in Korean Patients: Korean Registry of Acute Myocardial Infarction for Regional Cardiocerebrovascular Centers
Source: J Clin Med. 2021 Feb 1;10(3):498. doi: 10.3390/jcm10030498 (PMC7867023; doi:10.3390/jcm10030498)
Supplement: Supplementary file 1 [file jcm-10-00498-s001.pdf]

Table S1. Characteristics of the KRAMI-RCC patients by gender.

	Female (N = 3048)	Male (N = 8652)	Total (N = 11700)
Type of AMI			
NSTEMI	1953 (64.1%)	4668 (54.0%)	6621 (56.6%)
STEMI	1095 (35.9%)	3984 (46.0%)	5079 (43.4%)
Age (years), mean ± SD	74.0 ± 11.0	63.0 ± 12.4	65.9 ± 13.0
Type of health insurance			
public insurance	2737 (89.9%)	8107 (93.8%)	10844 (92.7%)
medical aid	288 (9.5%)	468 (5.4%)	756 (6.5%)
individual insurance	20 (0.7%)	72 (0.8%)	92 (0.8%)
Education level			
elementary school	1585 (52.0%)	1337 (15.5%)	2922 (25.0%)
middle school	426 (14.0%)	1207 (14.0%)	1633 (14.0%)
high school	536 (17.6%)	3210 (37.1%)	3746 (32.0%)
college	159 (5.2%)	2108 (24.4%)	2267 (19.4%)
graduate school	15 (0.5%)	314 (3.6%)	329 (2.8%)
unknown	325 (10.7%)	476 (5.5%)	801 (6.8%)
Risk factors and Medical history			
Current smoking	111 (3.6%)	2355 (27.2%)	2466 (21.1%)
High-risk alcohol useb	41 (21.2%)	1511 (40.7%)	1552 (39.7%)
Hypertension	1979 (64.9%)	4003 (46.3%)	5982 (51.1%)
Dyslipidemia	390 (12.8%)	1027 (11.9%)	1417 (12.1%)
Diabetes Mellitus	1144 (37.5%)	2370 (27.4%)	3514 (30.0%)
Previous PCI	433 (14.2%)	1181 (13.7%)	1614 (13.8%)
Previous CABG	37 (1.2%)	72 (0.8%)	109 (0.9%)
Previous dialysis	103 (3.4%)	194 (2.2%)	297 (2.5%)
Any cancer	150 (4.9%)	470 (5.4%)	620 (5.3%)
Symptom to first medical contact time (min), median (IQR)	121.5 [49.0;324.0]	84.0 [34.0;230.0]	90.0 [37.0;248.0]
Symptom to arrival time (min), median (IQR)	190.5 [86.0;425.0]	139.0 [61.0;311.5]	153.0 [65.0;336.0]
Symptom to arrival time			
<= 60 min	399 (17.1%)	1807 (24.9%)	2206 (23.0%)
60 - 180 min	722 (30.9%)	2449 (33.7%)	3171 (33.0%)
> 180 min	1217 (52.1%)	3004 (41.4%)	4221 (44.0%)
Door to device time at STEMI patients (min), median (IQR)	56.0 [46.0;67.0]	54.0 [44.0;65.0]	55.0 [44.0;66.0]
Type of transport to emergency room			
public ambulance	702 (23.0%)	2248 (26.0%)	2950 (25.2%)
private ambulance	1308 (42.9%)	3117 (36.0%)	4425 (37.8%)
cares or buses	1016 (33.3%)	3198 (37.0%)	4214 (36.0%)
walking	22 (0.7%)	89 (1.0%)	111 (0.9%)
Transfer from other hospitals	1651 (54.2%)	4349 (50.3%)	6000 (51.3%)

Performed PCI	2650 (86.9%)	7955 (91.9%)	10605 (90.6%)
Number of significant stenotic artery			
1	1524 (57.5%)	4780 (60.1%)	6304 (59.4%)
2	766 (28.9%)	2253 (28.3%)	3019 (28.5%)
3	360 (13.6%)	922 (11.6%)	1282 (12.1%)
Heart failure at arrival	383 (12.6%)	659 (7.6%)	1042 (8.9%)
Cardiogenic shock at arrival	271 (8.9%)	647 (7.5%)	918 (7.8%)
Cardiac arrest at hospital	239 (7.8%)	512 (5.9%)	751 (6.4%)
Stroke at hospital	29 (1.0%)	64 (0.7%)	93 (0.8%)
Atrial fibrillation at hospital	332 (10.9%)	634 (7.3%)	966 (8.3%)
In-hospital death	242 (7.9%)	413 (4.8%)	655 (5.6%)
All cause death from discharge during PH 3 months	81 (2.9%)	125 (1.5%)	206 (1.9%)
All cause death from discharge during PH 12 months	183 (6.6%)	301 (3.7%)	484 (4.4%)

NSTEMI: non-ST elevation myocardial infarction ; STEMI: ST elevation myocardial infarction; SD: standard deviation; IQR: Interquartile range; PCI: percutaneous coronary intervention; CABG: coronary artery bypass graft; PH: post-hospital
